# Case Report on Early Diagnosis of COVID-19

**DOI:** 10.1017/dmp.2020.66

**Published:** 2020-04-03

**Authors:** Yang Zhou, Le Yang, Ming Han, Minqiang Huang, Xuedong Sun, Weidong Zheng, Wei Han, Jinhong Wang

**Affiliations:** Emergency Department of Shenzhen University General Hospital, Shenzhen, Guangdong, China; Clinical Lab of Shenzhen University General Hospital, Shenzhen, Guangdong, China; Medical Imaging Department of Shanghai Mental Health Center, Shanghai, PR, China

**Keywords:** COVID-19, CT, epidemiology, pneumonia, SARS-CoV-2

## Abstract

The coronavirus disease 2019 (COVID-19) outbreak in Wuhan, China, spread rapidly throughout China and gradually to some countries abroad. How is the development of an epidemic controlled? Early diagnosis is one of the important contents in prevention and control. COVID-19 patients with early mild pneumonia often lack typical evidence to make a definitive diagnosis. Based on the analysis of the cases of 4 patients, this article finds that early diagnosis requires a combination of epidemiology, clinical manifestations, imaging, and etiology, with particular emphasis on epidemiology history and chest computed tomography (CT) manifestations.

The COVID-19 outbreak occurred in December 2019 in Wuhan, China. According to a report in China, it was related to a set of pneumonia cases in a seafood market in Wuhan, South China.^1,2^ Epidemiology indicates that COVID-19 involves person-to-person transmission.^3^ With the spread of the epidemic, similar cases have appeared in some foreign countries,^4^ and the disease has been initially recognized in the process of treating patients in various medical centers. However, there are still many issues to be discussed for early diagnosis, especially for patients who are highly suspected for infection and lack etiological evidence. The report analysis of the 4 cases of patients with common COVID-19 is presented as follows.

## CLINICAL FEATURES

**FIGURE 1:**
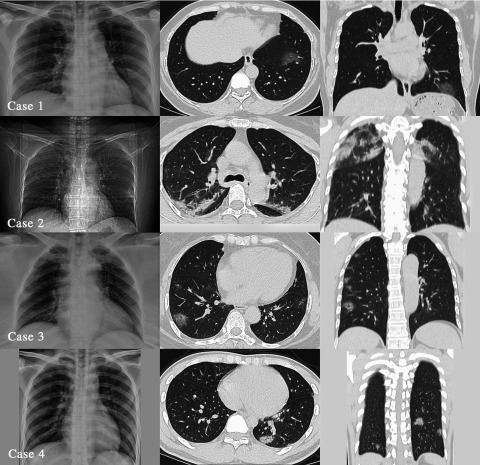
Four Patients’ Imaging Findings.

From January to February 2020, 4 patients with common COVID-19 were admitted and treated, and all 4 patients had a similar history from the Wuhan epidemic area and contact with patients confirmed with the disease. Three were female and 1 was male; 3 were young adults and 1 elderly. All 4 patients had fever not higher than 38.5°C. There were 2 cases of pharyngeal discomfort, 2 cases of dry cough, 1 case of productive cough, 1 case of muscle pain, 2 cases of headache, and 2 cases of fatigue (Table 1). None of the 4 patients showed nasal congestion, runny nose, vomiting, diarrhea, chest tightness, or shortness of breath. Chest examinations showed no positive signs, such as rhonchi or moist crackles, in both lungs. Peripheral blood leukocyte and lymphocyte decreased significantly in only 1 case. A pharyngeal swab SARS-CoV-2 ribonucleic acid (RNA) test was performed in 1 patient that was initially negative, 1 patient with twice negative, and 2 patients with initial positive. As for SARS-CoV-2 RNA pharyngeal swab test results, 1 was initial negative, 1 was twice negative, and 2 were initial positive (Table 2). Chest X-ray examinations were negative in all patients. Chest computed tomography (CT) scans showed multiple plaque, interstitial changes, and ground glass opacity in all 4 patients; a small amount of bilateral pleural effusion was observed in 1 patient (Figure 1; see Table 2).

**TABLE 1:**
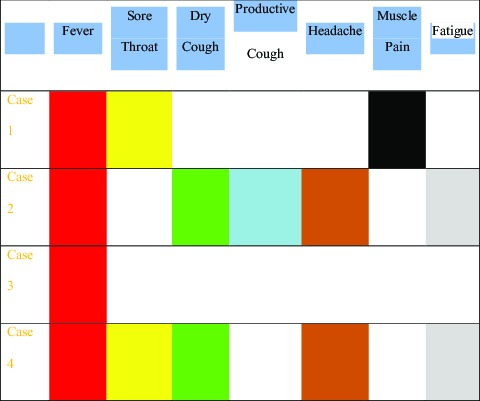
Clinical Symptoms of Patients

**TABLE 2:**
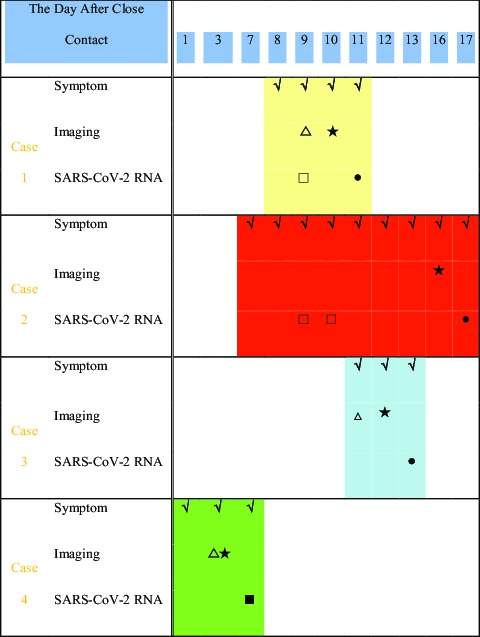
Results of Symptoms, Imaging, and SARS-CoV-2 RNA Test

*X-ray: △▴; CT: ☆★; Oropharynx: □▪; Nasopharynx: ○•; Solid: positive; Hollow: negative.

Patients’ imaging results included the following:

- **Case 1:** X-ray negative; CT: ground glass opacity of inferior lobe of left lung.
- **Case 2:** X-ray negative; CT: multiple patchy ground glass opacity in both lungs with a small amount of effusion on both lungs.
- **Case 3:** X-ray negative; CT: multiple patchy ground glass opacity on both lungs.
- **Case 4:** X-ray negative; CT: multiple patchy ground glass opacity on both lungs.

## DISCUSSION

According to these 4 confirmed cases, we found that (1) clinical manifestations of all patients were mild; (2) chest X-rays were negative and early CT findings detected severe manifestations in all cases; and (3) false negative and delayed positive results were found in SARS-CoV-2 RNA test of respiratory tract specimens. In summary, there are still many deficiencies in the understanding of the COVID-19 outbreak in Wuhan, China.

Cases 1 and 2 were negative in the first SARS-CoV-2 RNA test, and 1 case was still negative for the second test. In the end, the 2 patients were positive on Days 11 and 17 of the history of close contact and not right after symptoms appeared. This false-negative consideration may be related to the following factors: **(1) Disease development process:** The viral load carried by the patient is not enough to be detected positive; **(2) Collection position of respiratory secretions:** The current collection sites are mainly the oropharynx and nasopharynx. Both patients (Case 1 and Case 2) had oropharyngeal sampling when the test result was negative, and both had nasopharyngeal sampling when the test result was positive. It has been reported that the SARS-CoV-2 RNA detection rate from oropharyngeal sampling alone is lower than that from nasopharyngeal sampling.^5^ The reasons for the difference in results may be the environment of nasopharynx and oropharynx might influence the SARS-CoV-2 environment, the patients’ tolerance caused the swab to stay longer in the nasopharynx than in the oropharynx at the time of sampling (in addition, oropharyngeal sampling is more likely to cause droplet spray and occupational exposure); **(3) It may be related to the sampler’s technical level, inspection method, specimen kit, specimen preservation, and transportation.** ^6^ In summary, the SARS-CoV-2 RNA results obtained from single time, single site, and single sample are not reliable. The results of SARS-CoV-2 RNA from multiple times, multiple sites, and multiple specimens are of great significance for etiology diagnosis. Case 2 tested positive for SARS-CoV-2 RNA on the 17th day after close contact, which shows that, for some cases, especially those with occult infection, isolation for 14 days may not be enough.^7^ According to the official microblog of the People’s Daily in China, the 28th patient in Tianjin had twice negative SARS-CoV-2 RNA test results on the 6th day after the 1st day fever, and the 2nd fever on the 8th day. The test was still negative on the 9th day, and the 4th test on the 11th day was positive. Case 2 was similar to that reported by Tianjin Disease Control and Prevention Center.

There have been reports in China that SARS-CoV-2 RNA was detected in the stool of some patients with COVID-19, indicating that COVID-19 transmission is likely through fecal oral transmission.^8^ According to reports, SARS-CoV-2 can enter host cells through the cell receptor angiotensin-converting enzyme II (ACE2).^9^ ACE2 is not only highly expressed in alveolar type II cell, human esophageal epithelial cells, and stratified epithelial cells, but also in absorptive enterocytes cells from the ileum and colon.^10^ This study, through the bioinformatics analysis of single-cell transcriptomes, concluded that the digestive tract may be a potential pathway for SARS-CoV-2 infection. This is likely to be the cause of digestive symptoms in some patients with COVID-19. **In summary, whether the early diagnosis and follow-up of SARS-CoV-2-infected patients require the detection of SARS-CoV-2 RNA in feces, urine, and other excreta or body fluids needs further study**.

A prominent feature of the 4 patients was that the clinical signs and symptoms were inconsistent with the imaging: Signs and symptoms were mild, including normal pulse oxygen saturation, whereas the CT images showed that patients’ conditions were severe. According to the clinical signs and symptoms, all 4 cases were common mild cases, but the chest CT showed viral pneumonia, such as different levels of multiple patchy ground glass opacity and interstitial changes, and even 1 case had bilateral pleural effusion. However, all patients underwent a chest X-ray examination before a CT examination, and no positive results were found. **Therefore, for suspected patients,^7^ especially those with no evidence of etiology, we cannot always evaluate the lung condition comprehensively and objectively through a chest X-ray examination, so that misdiagnosis happens and affects treatment. Therefore, an early chest CT examination is relatively reliable, which may reduce the rate of a missed diagnosis. The value of quantitative analysis of chest CT images for the diagnosis of this disease needs to include more cases for further research**.

## CONCLUSION

Four patients had a clear epidemiology history, whose clinical manifestations were only fever, sore throat, cough, and other mild symptoms. Peripheral white blood cells and lymphocytes did not reduce significantly, early pathogenic tests results were negative, chest X-ray results were negative, and chest CT images showed significant changes in pneumonia. Early diagnosis of this type of patients cannot be limited to 2 negative SARS-CoV-2 RNA tests, according to which the disease cannot be excluded. The combination of epidemiology, clinical manifestations, imaging, and etiology are required to reduce the rate of a missed diagnosis, and special attention should be paid to epidemiology history and chest CT manifestations. **However, it may be unpractical to provide a full coverage of lung CT scans in early diagnosis due to a shortage of medical resources in some low-income countries: a possibly long wait, the expense concern, and so on will make case-confirming and treatment overdue. Given such concerns, chest plain film is advised to be an important reference for dynamic observation. In short, for early diagnosis of COVID-19, what must be done should always concur with what is available**.
